# Deep Reinforcement Learning Multi-Agent System for Resource Allocation in Industrial Internet of Things

**DOI:** 10.3390/s22114099

**Published:** 2022-05-28

**Authors:** Julia Rosenberger, Michael Urlaub, Felix Rauterberg, Tina Lutz, Andreas Selig, Michael Bühren, Dieter Schramm

**Affiliations:** 1 Bosch Rexroth AG, Automation and Electrification Solutions, 97816 Lohr am Main, Germany; julia.rosenberger@boschrexroth.de (J.R.); ask.michael.urlaub@outlook.com (M.U.); felix.rauterberg@boschrexroth.de (F.R.); tina.lutz@boschrexroth.de (T.L.); andreas.selig@boschrexroth.de (A.S.); 2 Westfälische Hochschule, 46395 Bocholt, Germany; michael.buehren@w-hs.de; 3 Faculty of Engineering, University of Duisburg-Essen, 47057 Duisburg, Germany

**Keywords:** deep reinforcement learning, multi-agent system, Industrial Internet of Things, load balancing, resource allocation, dynamic network

## Abstract

The high number of devices with limited computational resources as well as limited communication resources are two characteristics of the Industrial Internet of Things (IIoT). With Industry 4.0 emerges a strong demand for data processing in the edge, constrained primarily by the limited available resources. In industry, deep reinforcement learning (DRL) is increasingly used in robotics, job shop scheduling and supply chain. In this work, DRL is applied for intelligent resource allocation for industrial edge devices. An optimal usage of available resources of the IIoT devices should be achieved. Due to the structure of IIoT systems as well as security aspects, multi-agent systems (MASs) are preferred for decentralized decision-making. In our study, we build a network from physical and virtualized representative IIoT devices. The proposed approach is capable of dealing with several dynamic changes of the target system. Three aspects are considered when evaluating the performance of the MASs: overhead due to the MASs, improvement of the resource usage of the devices as well as latency and error rate. In summary, the agents’ resource usage with respect to traffic, computing resources and time is very low. It was confirmed that the agents not only achieve the desired results in training but also that the learned behavior is transferable to a real system.

## 1. Introduction

**Figure 1:**
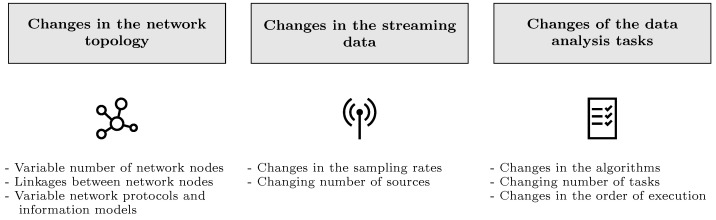
A systematic overview of the dynamic changes in IIoT networks.

In the last years, digitization and the Internet of Things has arrived in industry. It led to the fourth industrial revolution and, in addition to smart manufacturing and cyber-physical systems, the Industrial Internet of Things (IIoT) evolves [1]. Due to the new organization design principles in Industry 4.0 [1] and new business models, especially an increasing number of data-driven business models, the demand for edge computing is growing. Data analysis close to the data acquisition, processed on so-called edge devices for low latency and more data security, gains relevance compared to the currently prevailing cloud-based approaches [2]. An optimal usage of the generally scarce resources, namely CPU, RAM and memory, of edge devices, as well as bandwidth, is needed. Each device itself is very limited, but making optimized use of all available resources of large IIoT networks can push data analysis and other computing tasks to the edge. As the IIoT has the structure of a dynamic mesh net, one of the most important prerequisites is the capability to handle dynamic changes. In this work, a systematic overview of possible dynamic changes in IIoT networks is given (see Figure 1). They are divided into three groups. The first group includes the most obvious changes, i.e., changes in the network topology like linkages and network nodes. The two other groups consider changes from the perspective of the purpose of the network, namely changes in the transmitted data and in the applications carried out. They are also considered increasingly relevant for the next generation of distributed stream processing systems. As the fourth generation combines processing on edge and cloud, it is expected that the main processing in the fifth generation will run in the edge.

To maximize edge computing on IIoT network devices while taking into account load balancing across the network, a system for intelligent resource allocation is proposed that meets the following requirements.

- RQ1: The system should be adaptive to dynamic changes in the network.
- RQ2: Data loss due to overload has to be avoided.
- RQ3: No additional hardware should be required for the resource allocation.
- RQ4: The maximum number of different computing tasks is not specified and must match current and future requirements.
- RQ5: The system should be agnostic for different sensor signals.
- RQ6: The system should be able to handle streaming data processing tasks.
- RQ7: The parameterization effort should be low.

In large decentralized and dynamic systems, static programming is no longer sufficient. Modern approaches based on machine learning are said to be a more promising solution. In particular, Reinforcement Learning (RL) systems that learn via trial and error are suited for problems to be solved by sequential decision-making. So far, RL is typically applied to tasks in the fields of gaming, logistics, robotics and routing [3].

Chen et al. [4] identify the IIoT as one of the main scopes of application of Deep Reinforcement Learning (DRL). In the context of enabling edge computing, the computing tasks to be performed, especially data processing, can be intelligently allocated by RL agents according to the available resources of each network participant and the available bandwidth for transmission. Thus, available resources can be optimally utilized and further computations can increasingly be executed even on resource-limited devices. Different architectures, namely single-agent RL, centralized multi-agent reinforcement learning (MARL) and fully decentralized MARL, are compared and result in the latter to be the preferred approach. This article describes a possible implementation and its evaluation of the very basic idea originally sketched in [5]. As proposed in [5], each device runs its own agent to allocate its resources, and the decision-making takes place sequentially. This study differentiates from the previous study in the change to static-sized state and action spaces, the change of the observed resources (bandwidth, CPU, partly RAM but not hardware memory), the detailed description of the implementation, the comparison of different architectures and the evaluation. In [5], neither bandwidth allocation nor dynamic changes in the number of network participants and network linkages were taken into account. The main contributions of this study can be summarized as follows:

1. A systematic overview on dynamic network environments and approaches for handling different changes during runtime are presented.
2. A MARL system for intelligent allocation of IIoT computing resources is described.
3. A MARL system for intelligent bandwidth allocation is described.
4. Enabling edge computing in IIoT through cooperation of two independent MASs for resource allocation is demonstrated and evaluated.
5. A comparison of different architectures of DRL-agent systems is drawn.

The remainder of this article is organized as follows: Section 2 describes the state of the art of MARL for resource allocation in Industry 4.0, followed by an introduction of the relevant backgrounds of RL in Section 3. In Section 4, the detailed description of the proposed decentralized agent systems as well as their interactions are introduced. It is followed by comparisons of different possible architectures for problem–solution. Section 5 contains the validation focusing the low complexity for implementation of MASs in IIoT, the resource allocation itself and the ability to handle changes in topology. Section 6 concludes with a discussion and outlook for future work.

## 2. Related Work

Several RL systems for the optimization of different resources, e.g., production machine load, energy, computational load or bandwidth, with different optimization goals, e.g., energy efficiency, latency or load balancing, are already presented in literature. This section summarizes existing work on single- and multi-agent RL approaches for optimization of resource usage with focus on industrial applications.

In the industrial context, job shop scheduling (JSP) is one of the relevant tasks that is increasingly solved by RL systems. Wang et al. [6] use DRL for dynamic scheduling of jobs for a balanced machine utilization in smart manufacturing. In [7,8], two examples for energy optimization in cyber-physical production systems are presented and, in [9], real-time requirements for JSP fulfilled by a system of heterogeneous agents.

In the context of IIoT networks, the most common way to handle the resource limitations are computation offloading and mobile edge computing. In [4], Chen et al. provide a method for single-agent based DRL for dynamic resource management for mobile edge computing with respect to latency, energy efficiency and computation offloading for industrial data processing. For task offloading in IoT via single-agent DRL, a couple of further examples [10,11] exist already, as well as a few approaches based on multi-agent DRL [12,13,14]. For IoT networks in general, Lui et al. [12] propose a decentralized MARL for resource allocation that is used for the decision about computation offloading to a local server. Considering the specific challenges in industry, a MAS for computation offloading is presented in [13] but, different from the work of Liu et al. [12], using DRL. In contrast to our work, mobile edge computing applications generally offload tasks to an edge server instead of resource-limited IIoT edge devices. The main differences of our solution to these task offloading optimization problems are, firstly, the limited computing resources of the IIoT devices instead of offloading to the nearly unlimited servers in the fog and, secondly, the assumed infinity of the streaming data processing tasks; thus, metrics like the absolute number of CPU cycles for task execution are not known a priori. These challenges of distributed streaming data processing instead of finite task processing are considered in [15]. However, the solution is based on single-agent DRL and is not suitable to solve the challenges of dynamic networks due to the predefined size of the action space, which depends on the number of machines. A more flexible approach for data stream processing using MAS is presented in [16], using model-based RL for intelligent resource utilization.

Apart from JSP and computation offloading, a third field of application, communications and networking, is of increasing relevance [3]. DRL is a key technology for IIoT and, according to Chen et al. [4], resource optimization in industrial wireless networks is one of the main application fields. It is to differ between two main challenges in the context of communication resource optimization: firstly, resource allocation, i.e., channel allocation, frequency band choice, etc., and secondly, routing problems that are solved by finding the best transmission way through the network for a defined source and destination. In the context of resource allocation, the focus is on intelligent choice of communication parameters, e.g., frequency band, considered by Ye et al. [17] for vehicle-to-vehicle communication, where each vehicle or vehicle link represents an agent, and by Li and Guo [18], who use MARL for spectrum allocation for device-to-device communications. Gong et al. [19] present a multi-agent approach for minimizing energy consumption and latency in the context of perspective 6G industrial networks. The agents decide about task scheduling, transmission power and CPU cycle frequency.

Instead of allocating the resources for transmission, the choice of the best transmission path is the goal of RL systems for routing. For single-agent approaches, the agent chooses the whole path from source to sink [20]. Liu et al. [21] present a DRL routing method. The path is chosen under consideration of different resources like cache and bandwidth. To provide more scalability, MARL systems are often the preferred solution for decentralized applications. Thus, the systems are based on multi-hop routing as shown in [22,23,24]. These approaches differ from our work as the destination is known in advance, while our system searches for a suited destination for the processing. Nevertheless, the next-hop approach is still transferable assuming that the next neighbor could be a suitable destination.

Especially in case of wireless sensor networks and energy-harvesting networks, where energy is the most limited resource, DRL applications increasingly focus on energy usage optimization as it is limiting both computation and communication [22,25,26,27].

Another RL application field of high interest is load balancing. In the recent work of Wang et al. [28], DRL is used for latency improvement and load balancing for 5G in IIoT in a federated learning architecture. According to [29], most existing load balancing solutions are of centralized structure in decision-making and, thus, limited in effectiveness in large networks. The authors propose a multi-edge cooperation but still stick to a single-agent approach. MARL for load balancing is examined in [30] for controller load in software-defined networks. The problem definition is single objective as in [31], where MARL enables a load-balancing control for smart manufacturing. However, the cloud assistance needed contradicts our requirements. Load balancing is not an explicit goal of our work but is also indirectly covered by the proposed system presented in this work. Due to the goal of maximizing usage of the available resources and the assumption that the resources are always too scarce for the computational and transmission needs, resource usage over the whole network is expected to be balanced around the specified threshold.

The main objective, the combined optimization of both computing and communication resource allocation, is of great novelty, especially in the context of the IIoT. Thus, few studies [11,14] have considered this optimization potential so far. Our proposed system of two interacting MASs based on DRL differentiates these two approaches as follows:

- Architecture: In contrast to the proposed fully decentralized system, the existing approaches are either a single-agent [11] or a centralized MAS [14].
- Dynamic changes: Due to the centralized architectures, the adaptivity to dynamic changes is not given in the existing studies.
- Field of application: Only one of the existing algorithms is developed for application in industry [14].
- Data: Both existing algorithms are not suited for resource allocation for streaming data processing tasks.
- Objective: The main objective in this work is to maximize the edge computing using available edge resource rather than minimizing routing and computation delays [11,14].

In summary, there is no work known that considers the allocation of IIoT edge device resources for streaming data processing tasks and further edge computations in dynamic IIoT networks. Furthermore, no approaches of two interacting MARL systems in the context of network and computational resource allocation have been presented so far. The current relevance of the topic is evidenced by the high degree of topicality of the related work.

## 3. Background

**Figure 2:**
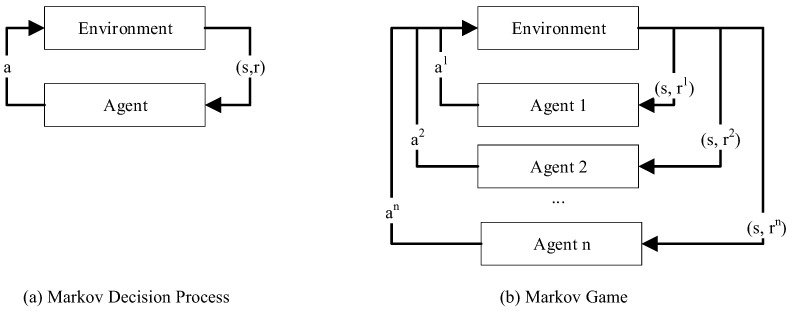
Comparison of the general models MDP (**a**), describing a single-agent-system, and MG (**b**), describing multi-agent systems, according to [32].

RL is a research field of machine learning in which an agent learns via trial and error. As shown on the left side of Figure 2, the single agent takes an action *a* depending on the current state $s_{t}$ of the environment and receives a reward *r*. This is mathematically described by the Markov Decision Process (MDP). The reward value depends on its current state $s_{t}$ and the next state $s_{t+1}$ the action $a_{t}$ puts him into, i.e., whether the chosen action is expedient for the goal achievement or not. With the long-term goal of maximizing the discounted reward $V^{\pi }\left(s_{T}\right)$, the agent tries to learn an optimal policy $\pi^{*}$, i.e., the mapping of current state and action for the best way to solve his mission. On the right side, the analogous structure of a system with more than one agent, i.e., a MAS, is illustrated. For MAS, the Markov Game (MG) can be used for mathematical description instead of the MDP.

### 3.1. Mathematical Preliminaries

In general, the simplest way to describe an RL system is the above-mentioned MDP. According to [32,33], the MDP is defined as 5-tupel (S,A,P,R,γ) with

- S: Set of states, s∈S;
- A: Set of actions, a∈A;
- P: Transition function, P:S;×A×S→[0,1];
- R: Reward function, R:S×A;×S→R;
- γ: Discount factor, γ∈[0,1).

The transition function *P* describes the probability that the environment passes at time *t* due to action $a_{t}$ from state $s_{t}$ into $s_{t+1}$, where applies $s_{t+1}∼P\left(s_{t+1}|s_{t},a_{t}\right)$. For this transition, the agent receives a reward *r* according to $R(s_{t},a_{t},s_{t+1})$. Based on the policy π, the agent chooses an action $a_{t}∼\pi \left(a_{t}|s_{t}\right)$ [32,34]. If the transition function and the reward function are explicitly known, e.g., by a model, the optimal policy could be found with one of the standard procedures, e.g., value iteration or policy iteration. Without this prior knowledge, model-free RL methods are to be applied [32,35].

The MDP assumes the environment to be fully observable, i.e., the observation space Ω is equal to the state space *S*. If only parts of the environment can be observed by the agent, a generalization of the MDP, the so-called partially observable MDP (POMDP), applies, which is defined by the 7-tupel (S,A,P,R,Ω,O,γ) [32,33]. The 7-tupel extends the MDP as follows:

- Ω: Set of observations, o∈Ω;
- O: Observation function, O:S×A×Ω→[0,1].

The observation function describes the probability that the agent has the observation $o_{t}$ at the time *t* when the environment changes due to action $a_{t}$ from $s_{t}$ in $s_{t+1}$.

As the MDP and POMDP are only suited for single-agent setups, further generalizations have to be considered for the description of the interaction of more than one agent with the environment. For a mathematical description of MAS, the MG, also called Stochastic Game (SG) [36], is used instead of the MDP. According to [32], the MG is defined by the 6-tupel $\left(N,S,{\left\{A^{i}\right\}}_{i\in N},P,{\left\{R^{i}\right\}}_{i\in N},\gamma \right)$. It applies:

- N: Set of all agents, N={1,…,n} and n>1;
- S: Set of all states, s∈S;
- $A^{i}:$ Set of all actions of the; agents *i*, $A:=A^{i}\times \ldots \times A^{n}$ and a∈A;
- P: Transition function, P:S×A×S→[0,1];
- $R^{i}:$ Reward function of the agent *i*, $R^{i}:S\times A\times S\to R$;
- γ: Discount factor, γ∈[0,1).

Each agent *i* has the goal to find its optimal policy $\pi^{i}$ with $a_{t}^{i}∼\pi^{i}\left(a_{t}^{i}|s_{t}\right)$. Depending on the state $s_{t}$ at the time *t*, each agent *i* takes an action $a_{t}^{i}$ simultaneously. The reward function $R^{i}\left(s_{t},a_{t}^{i},s_{t+1}\right)$ rates the transition to $s_{t+1}$. The transition function $P(s_{t}|a_{t}^{i},s_{t+1})$ describes the probability of this state transition. Figure 2 compares the MDP and MG.

Analogous to the POMDP for single agents, the POMG is a generalization of the MG that considers the partial observability of real-world applications and is defined, according to [37], by the 8-tupel $(N,S,{\left\{A^{i}\right\}}_{i\in N},P,$${\left\{R^{i}\right\}}_{i\in N},{\left\{\Omega^{i}\right\}}_{i\in N},{\left\{O^{i}\right\}}_{i\in N},\gamma )$. It extends the MG as follows:

- $\Omega^{i}:$ Set of all observations of the agent *i*, $\Omega :=\Omega^{i}\times \ldots \times \Omega^{n}$ and o∈Ω;
- $O^{i}:$ Observation function, $O^{i}:S\times A\times \Omega \to \left[0,1\right]$.

The observation function $O^{i}\left(o_{t+1}^{i}|a_{t}^{i},s_{t+1}\right)$ describes the probability for the observation $o_{t+1}^{i}$ at the time *t* for agent *i*, when the environment passes from $s_{t}$ into $s_{t+1}$ due to action $a_{t}^{i}$.

Under the assumption that all agents act simultaneously and are homogeneous, and thus interchangeable, and consequently have the same reward function, a fully observable system can be classified as a so-called Team Game [32], also called multi-agent MDP (MMDP) [38], which is a special case of the MG. This is further generalized by the decentralized POMDP (Dec-POMDP) that is similar to the MMDP but where agents only partially observe the entire state [38].

The mathematical model that considers a sequential—instead of simultaneous—decision-making of the agents is the Agent Environment Cycle (AEC) game [39]. In [37], Terry et al. prove that, for every POMG, an equivalent AEC game exists and vice versa. Thus, the methods AEC game and POMG are equivalent. The 11-tupel $(N,\;S,\;{\left\{A^{i}\right\}}_{i\in N},\;{\left\{T^{i}\right\}}_{i\in N},\;P,\;{\left\{R^{i}\right\}}_{i\in N}$, ${\left\{R^{i}\right\}}_{i\in N},\;{\left\{\Omega^{i}\right\}}_{i\in N},\;{\left\{O^{i}\right\}}_{i\in N},\;\gamma ,\;v)$ defines the AEC game according to [37], extended by the discount factor for the purpose of unification to previous described processes. The following adaptions are made in comparison to the POMG definition:

- $T^{i}:$ Transition function of the agents, $T^{i}:S\times A_{i}\to S$;
- P: Transition function of the environment, P:S×S→[0,1];
- $R^{i}:$ Set of all possible rewards for agent *i*, $R^{i}\subseteq R$;
- $R^{i}:$ Reward function for agent *i*, $R^{i}:S\times N\times A\times S\times R^{i}\to \left[0,1\right]$;
- v: Next agent function, v:S×N×A×N→[0,1].

At time *t*, agent *i* has an observation $o_{t}^{i}$ with the probability of the observation function $O^{i}$ and consequently takes the action $a_{t}^{i}$. It is to differentiate between two cases for status update from $s_{t}$ to $s_{t+1}$. For environment steps, (i=0) applies that the next state $s_{t+1}$ is random and occurs with the probability of the transition function *P*. Otherwise, for agent steps i>0, a deterministic state transition according to the transition function $T^{i}$ takes place [39]. Afterwards, the next agent $i^{\prime }$ with the probability of the next-agent function $v(i^{\prime }|s_{t},i,a_{t}^{i})$ is chosen. The reward function $R^{i}\left(r|s_{t},j,a_{t}^{j},s_{t+1},r\right)$ gives the probability that agent *i* receives the reward *r* if the action $a_{t}^{j}$ of agent *j* leads to the transition from $s_{t}$ to $s_{t+1}$.

**Figure 3:**
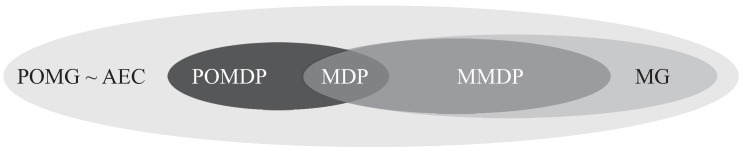
Venn diagram of the decision processes according to [38]. According to the diagram: MDP ⊂ MMDP ⊂ MG ⊂ POMG and MDP ⊂ POMDP ⊂ POMG and POMG ∼ AEC.

Figure 3 clarifies the described relations of the decision processes in a venn diagram.

MASs (n>1) can be further distinguished by the following criteria:

- Timing of actions: sequential or simultaneous decision-making.
- Reward function: unique or shared reward function.
- Agent types: homogeneous or heterogeneous agents.
- Interaction: cooperative or competitive behaviour.
- Training architecture: central training + central execution (CTCE), central training + decentral execution (CTDE), decentral training + decentral execution (DTDE).

### 3.2. Reinforcement Learning Algorithms

As the execution of DRL systems only depends on the trained policy, i.e., deep neural networks, the training of the neural networks requires further attention. The learning approaches are to be distinguished by the following criteria:

- Model: Model-based approaches have knowledge about the transition dynamics $P(s_{t+1}|s_{t},a_{t})$, so-called models [40]. Model-free approaches cannot access this knowledge. It is to highlight that both value and policy iteration algorithms are model based, as they are required to compute the Bellman operator [32].
- Collection of experience: While in on-policy learning, the target policy is the same as the behavior policy that interacts with the environment to collect experience; in off-policy algorithms, the agent bases its decision on the behavior policy while the target policy is optimized [32].
- Optimization approach: While value-based approaches optimize indirectly by trying to optimize the value function, policy-gradient approaches optimize directly on the policy. A combination that makes use of the advantages of both are actor–critic algorithms.

### 3.3. Network Topology of the Industrial Internet of Things

The IIoT is one of the main components of the Industry 4.0 [1]. The network consists of a large number of participants, primarily industrial edge devices like smart sensors or control units, with limited resources, but it can also have linkages to private or public clouds and local servers. In general, its topology is assumed to be a mesh network. This means the participants have a variable number of linkages between each other but need not to be fully connected.

## 4. Methodology

### 4.1. Problem Definition

**Figure 4:**
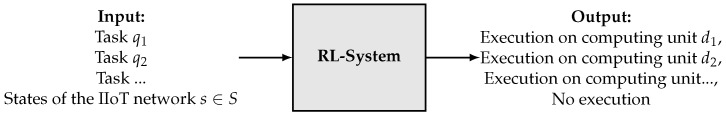
Abstraction of the problem statement.

The proposed DRL approach has to be able to allocate the execution of different edge computing algorithms to different IIoT network participants depending on the available resources. The problem is abstracted in Figure 4.

**Figure 5:**
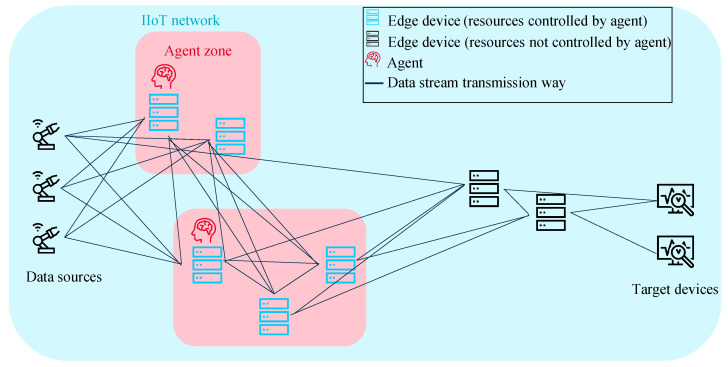
Schematic problem–solution approach on system level.

For the incoming tasks, it must be decided where they should be executed or, e.g., to avoid overloading, not to execute a task at all at this time. The decisions are made in dependence on the states of the IIoT network, i.e., the current available resources. In the context of this work, a task *q* can either be a pair of data stream and processing algorithm, e.g., anomaly detection [41] or data compression [42], or only a computing task that does not process data, e.g., a node in a distributed ledger technology network [43]. The proposed system optimizes the usage of multiple resources. The solution fulfills all above specified requirements RQ1-RQ7. In a first step, it is assumed that all computing units are capable and authorized to read and process all data. Furthermore, the agents decide only about CPU and bandwidth allocation; RAM usage is only considered as an observation so far. In Figure 5, the idea for solving the problem is schematically illustrated.

### 4.2. Proposed RL System

**Figure 6:**
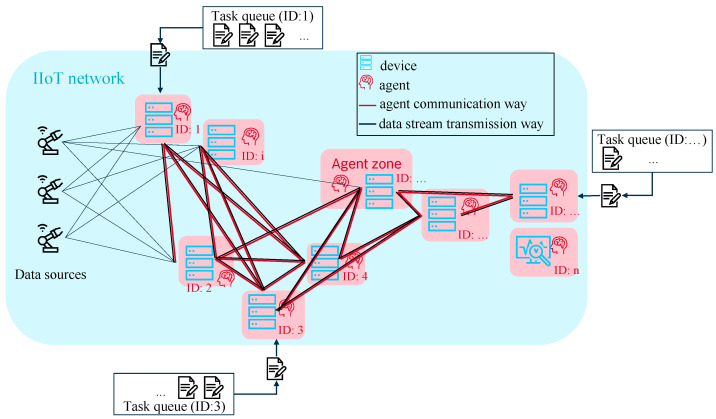
Schematic view on the fully decentralized multi-agent architecture.

**Figure 7:**
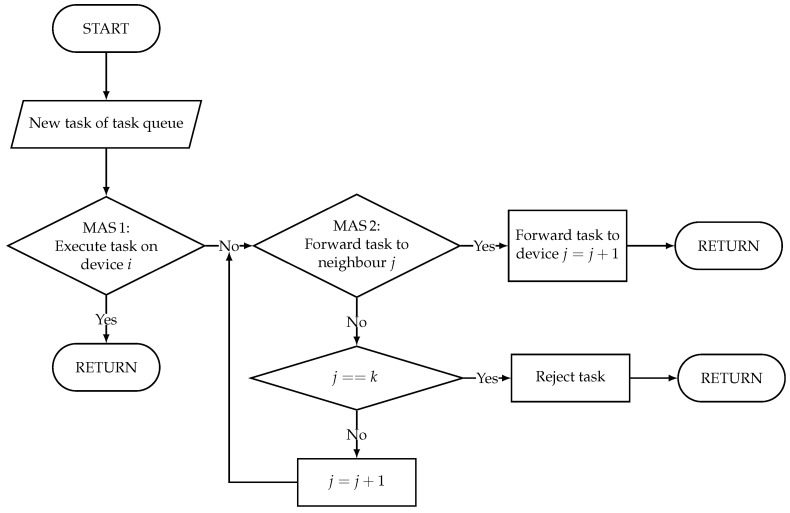
Interaction of MAS1 and MAS2 on one computing unit with *k* neighbors.

As both network and device resources are to be considered, the proposed solution for the described problem is an RL system that consists of two agent systems, one for the allocation of device resources, subsequently abbreviated with MAS1, and one for allocation of the network resources, subsequently abbreviated with MAS2. Due to its advantage of maximum flexibility with respect to scalability and adaptivity to the number of network nodes, the fully decentralized approach is superior to centralized systems and is chosen for both agent systems. It is shown schematically in Figure 6. The interaction of these two cooperating MASs is described as flow chart by Figure 7.

The presented fully decentralized systems are capable of handling a dynamic changing number of network nodes and linkages, i.e., dynamic changes in the network topology (RQ1), as well as changes in the data sources and processing tasks as the number of tasks does not have to be specified in advance (RQ4, RQ5). Both states of the single edge devices and tasks influence the overall set of states and set of actions, i.e., input and output sizes of the neural net. Varying input and output sizes are very difficult to handle by neural networks. Instead of existing approaches like DeepSets [44], Pointer Networks [45] or sequence-to-sequence-framework [46], the structure of the RL system is adapted, and the problem is broken down to static input and output sizes for a single agent decision. The piece-wise decision-making via subdividing the system into the smallest units, i.e., one agent per device and one task to decide about, maximizes the flexibility of the system. Thus, each computing unit runs one agent of MAS1 to decide about the allocation of the resources of its own computing unit. The input size is kept static as the agents take one decision about one task after the other. To avoid data loss due to overload (RQ2), a threshold that should not be exceeded is defined. In our RL systems, the thresholds of CPU and bandwidth load are set to 80%. In addition, the priority of the tasks assigned by the agents is set lower than the main tasks, e.g., a PLC task, of the edge devices. This secures that the agent systems do not interfere with a stable operation of the industrial plants. To fulfill RQ3, the agents are designed to be of low complexity so that they can be run on the existing IIoT devices and no additional devices are needed. According to RQ4 and RQ5, different computing algorithm and data streams to process should be executed. As the tasks are not known a priori, the agents are not trained on a specific set of tasks to allocate resources for. Considering RQ6, the ability to handle streaming data processing tasks implies that the processing of a task is not temporary. Thus, the allocated resources are permanently occupied by an assigned task. The agents are not trained on finite tasks and get no information about the total computational load of a task. A low parameterization effort according to RQ7 is ensured as the proposed RL system is not designed for a specific production site but to be adaptive for different IIoT networks. Adaptivity with respect to structure is achieved by the same mechanism as for adaptivity to dynamic changes, i.e., the subdivision of the system into the smallest units. Adaptivity with respect to processing algorithms and transmission rates is achieved by the agents’ training setup where random values are generated for both computational effort of the tasks as well as available and required bandwidth. For the training of MAS2, minor noise is added to these. In addition, the base loads of the devices, i.e., simulation of a load generated by running their main task, are varied.

Characteristic for MMDPs, the agents within each system MAS1 and MAS2 are homogeneous and, therefore, action space $A^{i}$, state space $S^{i}$ and reward function $R^{i}$ are identical for all agents in one system. The decision-making takes place sequentially, which leads to the classification as AEC game. For efficiency enhancements, the policy training is centralized, but execution is still decentralized. On-policy model-free approaches are chosen. All agents have to cooperate to reach the overall goal. For cooperation, the agents communicate and share relevant information while the decision-making still takes place locally. In reward shaping, it is considered that, according to [47], (a) the reward function should reward the goal achievement (what-reward) and not predefine how to achieve the goal (how-reward), and (b) there should be credit assignment of local and global rewards.

For both systems, the resource allocation is not a permanent process. It is triggered by a new task *q* in task queue *Q*. A watchdog functionality is developed to handle overload, i.e., rejection of an executed task. There are two cases, one is computational resource overload, i.e., CPU load, and the other is network resource overload, i.e., bandwidth. Overload is identified if either the computing unit or the transmitting linkage reaches a certain number of times 100% load within a defined time range. The detection of overload triggers the rejection of the latest task in the task execution list of the respective device with CPU overload or bandwidth overload when transmitting to the next neighbor. The rejected task is returned the same way as it was forwarded. The path is included as metadata in the task execution list. When the task reaches its origin, i.e., the first device that was deciding about the task execution, it is appended to the end of the task queue of that device so that it can be assigned in a new game round again.

### 4.3. MAS1: Decentralized Multi-Agent System for Computational Resource Allocation

The agents in the system MAS1 pursue the goal of allocating resources for execution of computing tasks on edge devices under the condition to keep the device load under a defined threshold. As the agents can only add tasks but not remove them, the maximization of edge computing is indirectly encouraged. The same applies for load balancing. It is assumed that the desired edge computing tasks exceed the limited device resources. Thus, it is to expect that the load of all devices will settle around the threshold value. In the following paragraph, the MMPD for MAS1 is defined, followed by the training procedure of MAS1 described in Algorithm 1.

- **Environment**: The computing tasks should be run on different IIoT devices $d_{i}$. Thus, the IIoT network is the environment of the agents.
- **Set of agents**: In dependence of the network size, i.e., the number of IIoT devices *D*, the number of agents varies as $N^{MAS1}=D$. For multi-agent systems, N={1,2,…,n}andn>1.
- **Set of states**: The observation array of each agent consists of two partial states $s_{t}=\left\{s_{t}^{1},s_{t}^{2}\right\}\;\mathrm{with}\;0<s^{1},s^{2}\leq 1\;\mathrm{and}\;s\in S$. $s^{1}$ describes the observed CPU usage of $d_{i}$ and $s^{2}$, the average CPU usage of all agents, calculated as follows:
  $$s^{2}=\frac{1}{n}\sum_{i=0}^{n}s^{1,i}$$
- **Set of actions**: There are two discrete actions: execute computing task (a=1) or not (a=0). For action a∈A, A={0,1}.
- **Reward**: All agents have the same reward function.
  $$R\left(s,a\right)=R^{1}\left(s,a\right)=R^{2}\left(s,a\right)=\ldots =R^{n}\left(s,a\right)$$
  Exceeding a threshold or inactivity are penalized. The allocation of resources for data processing below the threshold is rewarded.   
  (1) $$R^{MAS1}\left(s,a\right)=\left\{\begin{array}{cc} \mathrm{Penalty}\;r=-10 & \mathrm{if}\;a=1\;\mathrm{and}\;s_{t+1}>\;\mathrm{threshold}\\ \mathrm{Penalty}\;r=-1 & \mathrm{if}\;a=0\;\mathrm{and}\;s_{t+1}<\;\mathrm{threshold}\\ \mathrm{Reward}\;r=1 & \mathrm{else} \end{array}\right.$$

**Algorithm 1** Training procedure of MAS1.
     **Input** Task queue $Q=q_{1},q_{2},\cdots ,q_{n},\cdots$
1:All agents i∈N observe states $s_{i,t}=\left\{s_{i,t}^{1},s_{i,t}^{2}\right\}$2:**while** Training steps t<Set number of training steps **do**3:    **for** All agents i∈N **do**4:        Pass state $s_{i,t}$ to neural net as input5:        Get action $a_{i,t}$ as output6:        Next agent: i=i+17:    Pass chosen actions of all agents to step-function8:    **for** All agents i∈N **do**9:        **if** Action $a_{i,t}=1$ **then**10:           Add task load to CPU load $s_{i,t+1}^{1}$ of device *i*11:           **if** $s_{i,t+1}^{1}>\;\mathrm{threshold}$ or length(*Q*) <N **then**12:               Set done-flag13:           Recalculate average CPU load $s_{i,t+1}^{2}$14:        **else if** Action $a_{t}=0$ **then**15:           Append end of task queue *Q* by rejected task $q_{i}$16:        Award reward for agent *i*17:        Agent observes new state $s_{t+1}$18:        i=i+119:        Query done-flag20:    Pass done-flags, observations $s_{t+1}$ and rewards *r* to training algorithm21:    Optimize policy, i.e., neural net22:    **if** done-flag is set **then**23:        End of training24:    Training steps: t=t+1


### 4.4. MAS2: Multi-Agent System for Bandwidth Allocation

Besides MAS1 for allocation of computational resources, this subsection describes the second system MAS2 that interacts with MAS1 and is responsible for intelligent tasks forwarding in dependence of available bandwidth. It replaces a hard-coded next-agent function *v* of the AEC game by an intelligent mechanism also based on DRL to avoid data loss due to bandwidth bottlenecks. The following overview defines the MMDP for MAS2, followed by Algorithm 2 that summarizes the training procedure.

- **Environment**: The environment is again the IIoT network.
- **Set of agents**: The number of agents is—analogous to MAS1—equal to the number of computing units in the IIoT $N^{MAS2}=D$.
- **Set of states**: The observation of each agent includes the maximum available and the used bandwidth ($s^{1},s^{2}$) between the agent device and the next neighbor device *j*, the average bandwidth to all *k* neighbor devices ($s^{3}$), the computing resources CPU and RAM of the neighbor device *j* ($s^{5},s^{6}$) and the expected required resources, i.e., bandwidth, CPU and RAM, of the task ($s^{4},s^{7},s^{8}$).
  $$s=\left\{s^{1},s^{2},\ldots ,s^{8}\right\}\;\mathrm{with}\;s^{1},s^{2},s^{3},s^{4}\in R^{+}\;\mathrm{and}\;0<s^{5},s^{6},s^{7},s^{8}\leq 1\;\mathrm{and}\;s\in S$$
  (2) $$\mathrm{and}\;s^{3}=\frac{1}{k}\sum_{j=0}^{k}s^{1,j}$$
- **Set of actions**: Transmit task to neighbor *j* (a=1) or do not transmit and decide for next neighbor j+1 (a=0). For action a∈A, A={0,1}.
- **Reward**: All agents have the same reward function.
  $$R\left(s,a\right)=R^{1}\left(s,a\right)=R^{2}\left(s,a\right)\ldots =R^{n}\left(s,a\right)$$
  Similarly to the MAS1, exceeding a threshold or inactivity are penalized. The allocation of resources for task forwarding below the threshold is rewarded. The reward consists of three parts $R^{MAS2}\left(s,a\right)=R_{1}^{MAS2}\left(s,a\right)+R_{2}^{MAS2}\left(s,a\right)+R_{3}^{MAS2}\left(s,a\right)$.
  (3) $$\begin{array}{c} R_{1}^{MAS2}\left(s,a\right)=\left\{\begin{array}{cc} \mathrm{Penalty}\;r=\;-200\;\mathrm{in}\;\% & \mathrm{if}\;s_{t+1}>\;\mathrm{threshold}\;\mathrm{and}\;a=1\\ \mathrm{Penalty}\;r=\;-25\;\mathrm{in}\;\% & \mathrm{if}\;s_{t+1}<\;\mathrm{threshold}\;\mathrm{and}\;a=0\\ \mathrm{Reward}\;r=\;\mathrm{bandwidth}\;\mathrm{usage}\;\mathrm{in}\;\% & \mathrm{if}\;s_{t+1}<\;\mathrm{threshold}\\ \mathrm{Reward}\;r=\;\mathrm{threshold}\;\mathrm{value} & \mathrm{else} \end{array}\right.\\ \;R_{2}^{MAS2}\left(s,a\right)=\left(1-s_{t+1}^{5}\right)\cdot 50\;\;\mathrm{with}\;s_{t+1}^{5}\;\text{-}\;\mathrm{CPU}\;\mathrm{load}\;\mathrm{of}\;\mathrm{neighbor}\;j\;\mathrm{at}\;t+1\\ \;R_{3}^{MAS2}\left(s,a\right)=\left(1-s_{t+1}^{6}\right)\cdot 50\;\;\mathrm{with}\;s_{t+1}^{6}\;\text{-}\;\mathrm{RAM}\;\mathrm{load}\;\mathrm{of}\;\mathrm{neighbor}\;j\;\mathrm{at}\;t+1\\ \; \end{array}$$

**Algorithm 2** Training procedure of MAS2.
     **Input** Task queue $Q=q_{1},q_{2},\cdots ,q_{n},\cdots$
1:Agents i∈N observe states $s_{i,t}$2:**while** Training steps t< Set number of training steps **do**3:    **for** All agents i∈N **do**4:        Pass state $s_{i,t}$ to neural net as input5:        Get action $a_{i,t}$ as output6:        Next agent: i=i+17:    Pass chosen actions of all agents to step-function8:    **for** All agents i∈N **do**9:        **if** action $a_{i,t}=1$ **then**10:           Add task load to bandwidth usage of respective linkage ($s_{i,t+1}^{2}$)11:           **if** $s_{i,t+1}^{2}>\max.\;\;\mathrm{bandwidth}$ **OR** length(*Q*) < *N* **then**12:               Set done-flag13:           Recalculate average bandwidth usage of all linkages in environment ($s_{t+1}^{3}$)14:        **else if** action $a_{t}=0$ **then**15:           Append end of task queue *Q* by rejected task $q_{i}$16:        Award reward for agent *i*17:        Agent observes new state $s_{i,t+1}$18:        *i* = *i* + 119:        Query done-flag20:    Pass done-flags, observations $s_{t+1}$ and rewards *r* to training algorithm21:    Optimize policy, i.e., neural net22:    **if** done-flag **then**23:        End of training24:    Training steps: t=t+1


### 4.5. Execution and Interaction of MAS1 and MAS2

Unlike the training process, in which every agent participates in each game round, in the execution of the MASs, not every agent is involved in every game round. In the execution system, we define that one game round lasts from the first decision of an agent $1^{MAS1}$ about a new pending task to an agent $i^{MAS1}$ choosing action a=1, i.e., decides to execute the pending task, or the rejection of task by an agent $i^{MAS2}$, i.e., the decision of not forwarding. The metadata of the task is extended by a list of involved agents, i.e., subsequently appending the path of the task through the network. If the task cannot be assigned for execution, it is sent back the entire path and is again appended to the end of task queue of the first agent connected to the data source. The interaction and execution of both systems MAS1 and MAS2, previously separately trained, is summarized in Algorithm 3.
**Algorithm 3** Interaction of MAS1 and MAS2.1:Next pending task $q_{i}$ in task queue $Q_{i}$ for device $d_{i}$2:Query states of device and neighbor devices3:**if** Agent $i^{MAS1}$ decides for execution (a=1) **then**4:    Agent triggers execution on device5:    Path of task is extended by Agent *i*6:    Metadata of task is added to local list of executed tasks7:    **done**8:**else if** Agent $i^{MAS1}$ decides against execution (a=0) **then**9:    Hand over task to agent $i^{MAS2}$10:    **for** Neighbors 1…k of device *i* **do**11:        **if** Agent $i^{MAS2}$ decides for forwarding to neighbor *j* **then**12:           Add task $q_{i}$ to task queue $Q_{j}$ for device $d_{j}$13:           Extend path of task by *j*14:           i=j15:           **go to** *top*16:        **else if** Agent $i^{MAS2}$ decides against forwarding to neighbor *j* **then**17:           **if** Neighbor j==k **then**18:               Task is rejected and removed from task queue19:               Task is sent back to first device $d_{i}$ according to task path20:               Append task to end of task queue21:               **done**22:           j=j+1

Due to the separate training, there is no way to award the agents with a global reward for their overall goal achievement. In the case of combining both systems already in the training procedure, a global reward is expected to optimize the learning behavior. We propose a global reward that depends on the successful execution of the task and the number of hops *h* between first and last agents’ device when game round is finished. It is awarded in the end of a game round. It is needed to add the number of hops as an additional observation in MAS1 to ensure a learning success. Adding the hops as observation in MAS2 is not expedient as the MAS1 agents decide about the execution on the respective edge device, and thus, there is the need for further transmission. A global reward might be shaped as described in Equation (4).

(4) $$R^{global}\left(s,a\right)=\left\{\begin{array}{cc} \mathrm{Reward}\;r_{1}^{global}=80\cdot 0.9^{h} & \mathrm{Decision}\;\mathrm{for}\;\mathrm{execution}\;\mathrm{of}\;\mathrm{task}\\ \mathrm{Penalty}\;r_{2}^{global}=-80\cdot 1.1^{h} & \mathrm{Rejection}\;\mathrm{of}\;\mathrm{task} \end{array}\right.$$

To avoid the loss of tasks, tasks may only be forwarded to devices controlled by an agent. In addition, it is relevant to check the permission of the device to receive and process the data. Both can be ensured by adding the needed information to the asset administration shell [48] of the Industry 4.0 components. In the vision of IIoT, the devices are addressable via the administration shell, which is comparable to a digital type plate with all relevant information about the software and hardware of the asset [49].

### 4.6. Comparison to Other Agent System Architectures

In addition to the proposed fully decentralized system, other architectures should be mentioned and compared. The following subsection describes a comparison of three architectures of RL agent systems for resource allocation differing in their expression of decentrality, i.e., a single-agent approach, a centralized multi-agent approach and the chosen fully decentralized system already described in Section 4.3. In a preceding study, all three approaches were evaluated and compared on the use case of computational resource allocation, resulting in the above-presented fully decentralized system to fit the specified requirements best. Table 1 summarizes the results in a qualitative manner.

#### 4.6.1. Single-Agent System

**Figure 8:**
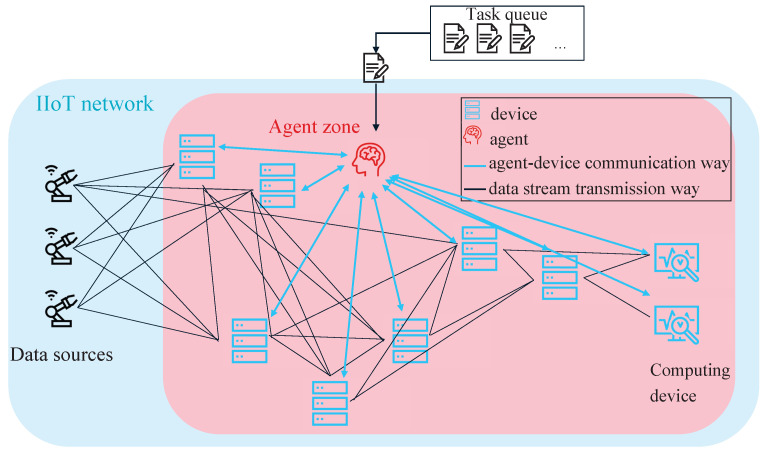
Schematic view on the architecture of the single-agent approach. The single agent has full view on the network and can communicate with all computing units. The agent receives the current CPU usage of the devices as states and either chooses a computing unit for task execution or no execution of the task. No execution would lead to appending the task again at the end of the task queue.

The first system is of full centralized structure with only one agent, illustrated in Figure 8. It is expected to make good decisions due to the full view on the network, i.e., the resource consumption of all network devices, and no uncertainties due to the decisions of other agents.

The single-agent approach is defined as follows:

- **Set of states**: The observed states include the percentage CPU loads $s^{i}$ of all computing units *N* in the network and the expected load of the task to allocate resources for $s^{0}$ with s∈SandS=|N+1|.
- **Set of actions**: The action space A=|N+1| covers all observed computing units *N* that can be chosen for task execution $a_{1},\;a_{2},\ldots ,\;a_{N}$ plus one additional action: to not execute the task at all $a_{0}$. The agent chooses one action a∈A per time step.
- **Reward**: The allocation of resources for data processing below the threshold is rewarded, while no allocation under or allocation above the threshold is penalized. For the reward function, R(s, a) applies:
  $$R\left(s,a\right)=\left\{\begin{array}{cc} \mathrm{Reward}\;r=20 & \mathrm{if}\;a_{t,j}=1\;\mathrm{and}\;s_{j,t+1}<\mathrm{threshold}\\ \mathrm{Penalty}\;r=-20 & \mathrm{if}\;a_{t,j}=1\;\mathrm{and}\;s_{j,t+1}>\mathrm{threshold}\\ \mathrm{Reward}\;r=20 & \mathrm{if}\;a_{t}=0\;\mathrm{and}\;\mathrm{all}\;s_{i,t+1}>\mathrm{threshold}\\ \mathrm{Penalty}\;r=-5 & \mathrm{if}\;a_{t}=0\;\mathrm{and}\;\mathrm{minimum}\;\mathrm{one}\;s_{i,t+1}<\mathrm{threshold}\\ \mathrm{Penalty}\;r=-10 & \mathrm{if}\;a_{t}=0\;\mathrm{and}\;\mathrm{all}\;s_{i,t+1}<\mathrm{threshold} \end{array}\right.$$

#### 4.6.2. Centralized Hierarchical MAS

**Figure 9:**
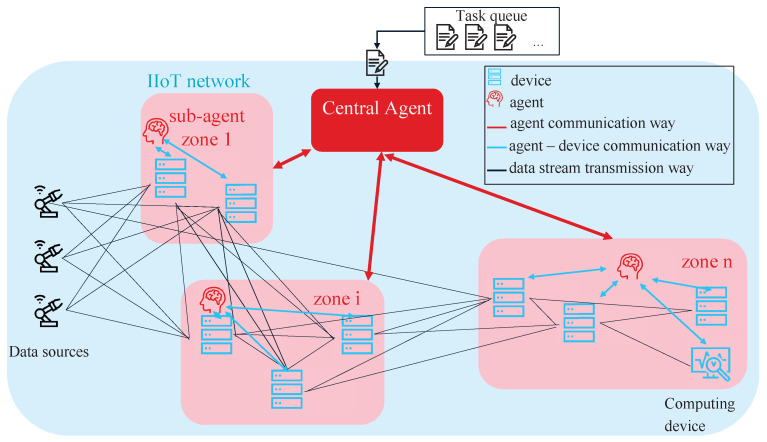
Schematic view on the architecture of the centralized multi-agent approach. The controller receives the incoming tasks to allocate resources for. It communicates with the sub-agents but not with the computing units directly. Depending on the states provided by the sub-agents, the central agent forwards the task to one of the sub-agents, which will further assign the task to one of its observed computing units.

The second approach is a centralized hierarchical MAS. It consists of one central agent, here called controller, that interacts with further decentralized agents, here called sub-agents, schematically illustrated in Figure 9. Thus, it is to distinguish between two different types of agents. The sub-agents cannot interact or communicate amongst each other. While all sub-agents are homogeneous, the central agent differs from them. In our implementation, each sub-agent is equivalent to the previous described single-agent approach. The MDP of the central agent is defined as follows:

- **Set of agents**: There is exactly one agent that acts as central instance.
- **Set of states**: The controller observes one state per sub-agent $i^{sub}$. Thus, the cardinality of the state space is $S=|N^{sub}+1|$ with s∈S. Each state $s^{sub_{i}}$ describes the average resource usage of all *m* devices $d^{sub_{i}}$ in the local environment observed by each sub-agent $i^{sub}$.
  (5) $$\;s^{sub_{i}}=\frac{1}{d^{sub_{i}}}\sum_{i=0}^{m}d_{CPU}^{i}$$
- **Set of actions**:The action space with the cardinality $A=|N^{sub}|$ covers all observed sub-agents that can be chosen for task forwarding.
- **Reward**: The controller-agent receives a reward for choosing the sub-agent with the lowest averaged computational load in its observed environment.
  $$R\left(s,a\right)=\left\{\begin{array}{cc} \mathrm{Reward}\;r=20 & \mathrm{if}\;\mathrm{action}\;a_{t}=a_{i}\;\mathrm{and}\;s_{t}^{sub_{i}}<s_{t}^{sub_{j}}\;\\ \mathrm{Penalty}\;r=-20 & \mathrm{else} \end{array}\right.$$

#### 4.6.3. Fully Decentralized MAS

The fully decentralized MAS corresponds to the system described in detail in Section 4.3. It consists of as many agents as devices in the network.

#### 4.6.4. Qualitative Comparison

This section summarizes the advantages and disadvantages of the different architectures. The biggest advantage of the single-agent approach is its full view on the states of the network and, thus, no uncertainties in decision-making. Since the computational resources for execution of the agent on edge devices are limited and the computational load depends on the size of the problem space, which, in this case, directly depends on the size of the network, it does not have unlimited scalability. Additionally, querying the states of all other devices in the network is time consuming. The centralized multi-agent solution improves as the load is shared among the sub-agents, which can also be seen as a hierarchical approach. The number of the controlled devices by the single-agent or controlled sub-agents by the controller agent is crucial for the number of inputs and outputs of the neural network. Thus, it has to be defined a priori, and adaptive changes in the number of network participants are not possible during runtime. As already described in Section 4.2, the fully decentralized approach is able to solve both scalability and adaptivity issues. One disadvantage compared to the two previously mentioned architectures is the limited observation space, since each agent only can observe its own devices’ states and the average load of the nearest neighbors. The communication overhead for the decentralized system cannot be defined in general terms, since it depends on the particular implementation. In our presented system, it is reduced to a minimum. In case of n:n communication between all agents, it would be significantly higher than in the other approaches. From the perspective of security aspects, the decentralized system prevails due to its fault tolerance. While, in our preferred solution, a fully decentralized MAS is chosen for its scalability and adaptivity, it should be emphasized that, depending on the network structure and requirements, the other two approaches may also prevail, as a fully observable system may achieve more optimal results.

Further optimizations in terms of scalability and adaptivity of the single and centralized agents might be achieved if the one-hop decision-making of the central agents is changed into stepping through all existing devices one by one.

## 5. Results

This section contains the results of the experiments evaluating the presented RL system to intelligently allocate resources for enabling edge computing in IIoT. The description of the experimental setup follows three subsections about the training phase and experiments evaluating the overhead of the agents themselves and the performance of the system in optimizing resource usage.

### 5.1. Experimental Setup

For the experiments, both MASs were implemented in Python 3.8.10 and were executed on a network of industrial control units to simulate an IIoT, namely physical ctrlX CORE from Bosch Rexroth with a 64-bit quad core ARM CPU, 1 GB RAM and 4 GB eMMC memory and virtual ctrlX COREs from Bosch Rexroth with a 64-bit quad core AMD CPU, 4 GB RAM and 4 GB eMMC memory, run as virtual machines on a laptop with an Intel Xeon E3-1503M v5 processor and 32 GB RAM. The operating system is Linux Ubuntu Core that requires the agents to be run as snaps. The network consists of one physical and six virtual devices and, thus, seven agents in each MAS. As network structure, the grid topology that was chosen is a special type of mesh network.

The following RL python libraries were used: The OpenAI Gym [51] is a python library from OpenAI [52] that enables episode-based RL. It allows an abstraction of a POMDP environment for a single agent. The library does not include functions for the agents. Analogous to the OpenAI Gym, the python library PettingZoo [53] (version 1.15.0) provides environments for MAS that represent an abstraction of AEC games [37]. Furthermore, the library includes standardized ways, using the python package SuperSuit (version 3.3.3), of a parallelized execution of the environments, which allows us to implement POMGs that are equivalent to AEC. A third library used is StableBaselines3 [54] (SB3, version 1.1.0), developed by the German Aerospace Center. It includes pretrained implementations of model-free RL methods for finding the optimal policies for the above-mentioned environments. For the snaps, the SB3 model was exported as a TensorFlow Lite model, which is explicitly suited for edge devices.

### 5.2. Training Results

The first part of evaluation considers the training phase. The systems MAS1 and MAS2 are trained separately as described in Algorithms 1 and 2, according to the parameterizations listed in Table 2.

**Figure 10:**
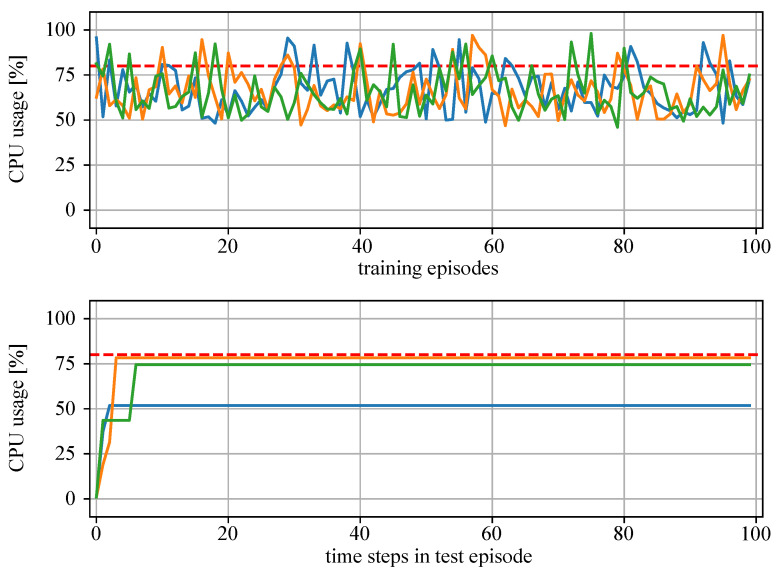
Results of the training and test of MAS1 with three agents (green, blue, orange lines: CPU usage of agent devices; red dotted line: threshold 80%). Above: CPU usage at the end of each training episode. Below: CPU usage at each time step of a single test episode.

**Figure 11:**
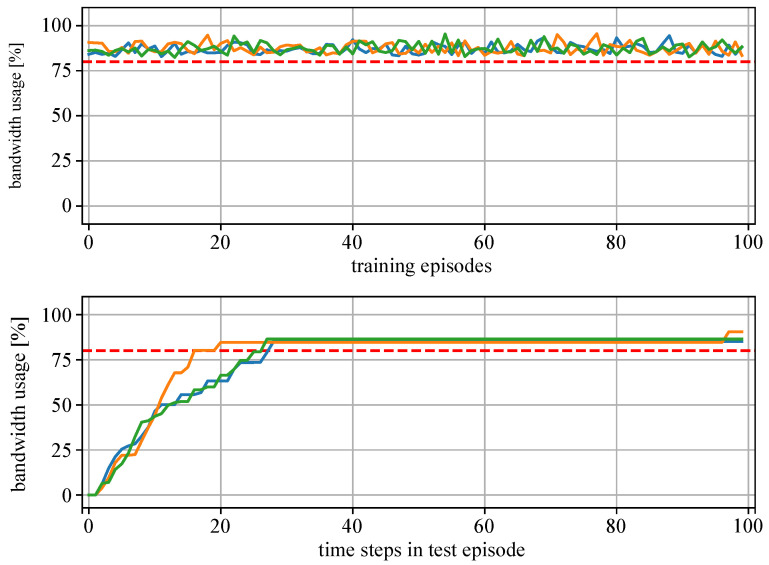
Results of the training and test of MAS2 with three agents (green, blue, orange lines: bandwidth usage; red dotted line: threshold 80%). Above: Bandwidth usage at the end of each training episode. Below: Bandwidth usage at each time step of a single test episode.

Figure 10 presents the training results of MAS1 and Figure 11, the results of MAS2. The upper plots show the CPU bandwidth usage at the last time step of the training episodes. The lower plots show the resource usage at each time step of the test episode. It can be seen that the training of both MAS1 and MAS2 agents (green, orange and blue lines) was successful as they learn to allocate resources for additional tasks up to the specified threshold value of 80% (red dotted line), without overloading the devices.

### 5.3. Evaluation of Resource Consumption

Secondly, the overhead due to the agent systems, i.e., the resource consumption by the agents themselves, was evaluated. Table 3 summarizes the results of measuring the resource consumption, i.e., CPU usage, RAM usage, traffic and memory, of the agents-snap, including a MAS1 and a MAS2 agent, executed on the physical control unit. The time required for the execution of the agents, i.e. the decision making itself, is summarized in Table 4.

The traffic overhead is generated by the communication between the agents to accomplish their task. It depends on the number of decisions and hops and consists of three communication contents: the description of the task, the agents’ observation query and the callback about the found route. This information is transmitted in JSON format. The traffic overhead has three causes and is calculated by adding up:

- the number of tasks forwarded times the size of the task description (∼217 bytes);
- the number of decisions about task forwarding times the size of states of the neighbor (∼50 bytes);
- the number of found routes times the number of hops times the callback information (∼149 bytes).

### 5.4. Evaluation of Performance

**Figure 12:**
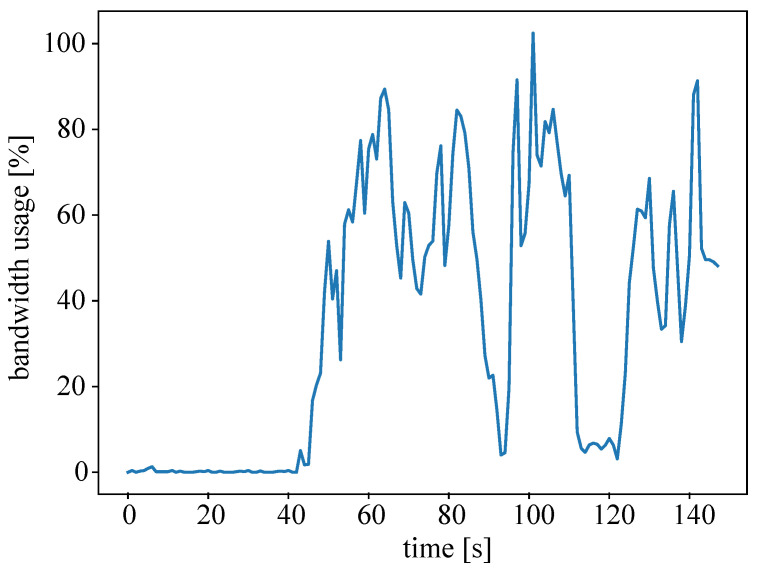
Exemplary bandwidth usage in the test environment with data streams of finite length allocated by MAS2.

For the last part of evaluation, the performance and benefit of the agent system, i.e., the optimization through intelligent resource allocation, was measured. Figure 12 illustrates an exemplary usage of bandwidth. In the experiments, the required task executions are finite, i.e., the data streams are also finite and end after a given period of time, to obtain more dynamics in the system. The results are similar to the results of the training phase (see Figure 11); thus, the agents’ good performance in the training environment is transferable to the test setup. The decision of the agent resulted in overload (100% peak) only one time.

The test system was able to allocate resources for task execution and, in 38 cases, suitable routes for task forwarding were found. Table 5 summarizes the latency and hops per route.

### 5.5. Limitations

The experimental evaluation presented is subject to the following limitations. A distinction is made between limitations of the proposed method itself and limitations of the experiments.

Limitations of the proposed method:

- Resources: Up to now, CPU load and bandwidth are considered for allocation. Further computing resources like RAM and hardware memory as well as energy are not considered. The algorithm is not explicitly optimized for energy-constrained networks such as wireless sensor networks.
- Priority: In this study, all computing tasks have the same priority, and priority is not yet considered by the agents.
- Permissions: In this study, all network participants are permissioned to read and process the data.
- Hyperparameter tuning: Hyperparameters are manually chosen. So far, no hyperparameter optimization methods have been applied.
- Overhead: If all devices are heavily loaded, the agent system is not able to allocate resources but causes additional load by forwarding requests.
- Data loss: Data loss is accepted until a route is found. Therefore, the approach is not suitable for applications where no lost bit is allowed.
- Action space: The agents decide whether or not to add load, but they cannot reduce load by terminating running tasks.
- Field of application: The training was intentionally very general so that the agents can be used in most industrial setups. Optimizing the policy to specific use cases, e.g., learning long-term dependencies, requires training the agents with specific data.

Limitations of the experimental evaluation:

- Implementation: The agents were implemented in the snap format, and Python is the chosen programming language. To improve speed, other programming languages should be taken into account. As a more generic format, the Open Neural Network Exchange (ONNX) format could be considered.
- Network topology: Only grid topology was evaluated. Since it is a form of mesh network and dynamic meshes are assumed to be the most complex topology, most cases should be covered by the experiments.
- Network size: The evaluation network was of small size compared to real industrial networks. Different and especially larger network sizes should be evaluated in order to be able to make a more precise statement about the full potential of the proposed algorithms.
- Field of application: The evaluation took place in a test setup of homogeneous control units.
- Adaptivity: Adaptivity to dynamic changes was not part of the experiments yet.
- Metrics: Computing and communication resource usage of the agents-snap as well as latency and hops of the successfully found routes were evaluated. Additional metrics such as the context of the overall resource usage in the network and the energy consumption of the agents are not yet considered.

## 6. Discussion

The proposed MARL-based approach for optimal edge resource usage consists of two interacting multi-agent DRL systems that are able to allocate computational power and bandwidth in dynamic IIoT networks to enable edge computing in the IIoT. The requirement of adaptivity to various dynamic changes is fulfilled, firstly, by the system structure subdivided into the smallest units and sequential decision-making for full adaptivity in the number of tasks, nodes and linkages and, secondly, by the training process not limited to a specific data stream or algorithms to compute for achieving the agents to act in a generic manner. A comparison of the three approaches with different degrees of decentrality is drawn, and the preferred architecture, a fully decentralized system, is selected under consideration of the specified requirements and network topology.

The suitability of RL-based resource allocation on resource-limited IIoT devices is confirmed by evaluation results. The experiments show that the proposed system is of low computational complexity, decision-making is very fast and the models are of small size; thus, it is suited for application on resource-limited IIoT devices. Both traffic overhead and latency between the first decision about a new task and the successful forwarding to an edge device that can compute the task depend on the overall CPU and bandwidth load in the network. Both increase when the resources are highly utilized, which complicates the resource allocation, and more decisions, hops and time are needed. While agent decision-making is very fast and takes only milliseconds, the overall latency needs to be improved as it is many times higher. This is caused by the number of decisions and the time span between the request of MAS2 agents and response of the neighbor’s available computing resources (states $s^{5},\;s^{6}$). To improve performance, the change to other programming languages like C or C++ should be considered.

In future work, experiments will be extended by evaluation of the success rate of task execution depending on the overall resource usage and performance measurements in dealing with dynamic network changes. Furthermore, the proposed system should be evaluated in a real IIoT network, e.g., a production site, with a higher number of nodes and linkages. In addition, a comparison to a manual, static task allocation would be helpful to be better able to classify the results. As most data processing is still done via cloud computing and there is not a state-of-the-art method for edge task allocation yet, it is difficult to draw this comparison. In a further step, the following aspects should additionally be considered: In MAS1, the state space could be extended by the resources RAM and hardware memory as well as the already needed hops. In MAS2, the priority of data or task as well as permissions for reading the data of the respective IIoT network participants should be considered. As mentioned above, the proposed global reward and a combined training of both systems would be interesting to evaluate as it is expected to improve results. A further improvement could be a managed degree of difficulty of processing an algorithm. The MARL agents could decide to execute the algorithm in a mode of heavy or light computational load.

## Figures and Tables

**Table 1:** Advantages and disadvantages of different agent system architectures.

	Single Agent	Centralized MAS	Decentralized MAS
Scalability	low	limited	high
Adaptivity	no	no	yes
Observation	full	partial	marginal
Communication load	medium	medium	low/medium/high *
Single point of failure	yes	yes	no

* Communication load in decentralized systems highly depends on the implementation (see [50]).

**Table 2:** Training parameters of MAS1 and MAS2.

Parameter	MAS1	MAS2
Total number of training steps	100,000	100,000
Training steps per episode	100	100
Learning rate α	0.003	0.003
Discount factor γ	0.99	0.99
RL algorithm	PPO	PPO
Number of agents	3	3

**Table 3:** Resource demand of the agents-snap measured on the physical control unit.

	CPU	CPU	Hardware	RAM ^a^	RAM ^a^
	avg. [%]	max. [%]	Memory [MB]	avg. [%]	max. [%]
Agents-Snap	0.81	6.60	53.8	4.6	4.7

^a^ RAM is only used for task queue and list of executed tasks incl. metadata of tasks.

**Table 4:** Run time of agents of MAS1 and MAS2.

Agent	$t_{\mathrm{avg}}\;\left[\mathrm{ms}\right]$	$t_{\max}\;\left[\mathrm{ms}\right]$
MAS1-Agent	0.74	1.16
MAS2-Agent	0.81	0.98

**Table 5:** Latency and hops per successful task forwarding.

Time ^b^ avg. [s]	Time ^b^ max. [s]	Hops *h* avg. []	Hops *h* max. []
4.84	11.87	1.84	3

^b^ Time span between first decision and successful routing to executing device. The values highly depend on the overall network load.

## Abbreviations

The following abbreviations are used in this manuscript:

AEC	Agent Environment Cycle
DRL	Deep Reinforcement Learning
IoT	Internet of Things
IIoT	Industrial Internet of Things
MARL	Multi-Agent Reinforcement Learning
MAS	Multi-Agent System
MDP	Markov Decision Process
MG	Markov Game
MMDP	Multi-Agent Markov Decision Process
POMDP	Partially Observable Markov Decision Process
POMG	Partially Observable Markov Games
SG	Stochastic Games
RL	Reinforcement Learning

## References

[1] Hermann M., Pentek T., Otto B. Design Principles for Industrie 4.0 Scenarios. Proceedings of the 2016 49th Hawaii International Conference on System Sciences (HICSS).

[2] Shi W., Cao J., Zhang Q., Li Y., Xu L. (2016). Edge Computing: Vision and Challenges. IEEE Internet Things J..

[3] Luong N.C., Hoang D.T., Gong S., Niyato D., Wang P., Liang Y.C., Kim D.I. (2019). Applications of Deep Reinforcement Learning in Communications and Networking: A Survey. IEEE Commun. Surv. Tutor..

[4] Chen Y., Liu Z., Zhang Y., Wu Y., Chen X., Zhao L. (2021). Deep Reinforcement Learning-Based Dynamic Resource Management for Mobile Edge Computing in Industrial Internet of Things. IEEE Trans. Ind. Inform..

[5] Rosenberger J., Urlaub M., Schramm D. Multi-agent reinforcement learning for intelligent resource allocation in IIoT networks. Proceedings of the 2021 IEEE Global Conference on Artificial Intelligence and Internet of Things (GCAIoT).

[6] Wang L., Hu X., Wang Y., Xu S., Ma S., Yang K., Liu Z., Wang W. (2021). Dynamic job-shop scheduling in smart manufacturing using deep reinforcement learning. Comput. Netw..

[7] Bakakeu J., Kisskalt D., Franke J., Baer S., Klos H.H., Peschke J. Multi-Agent Reinforcement Learning for the Energy Optimization of Cyber-Physical Production Systems. Proceedings of the 2020 IEEE Canadian Conference on Electrical and Computer Engineering (CCECE).

[8] Roesch M., Linder C., Bruckdorfer C., Hohmann A., Reinhart G. Industrial Load Management using Multi-Agent Reinforcement Learning for Rescheduling. Proceedings of the 2019 Second International Conference on Artificial Intelligence for Industries (AI4I).

[9] Luo S., Zhang L., Fan Y. (2021). Real-Time Scheduling for Dynamic Partial-No-Wait Multiobjective Flexible Job Shop by Deep Reinforcement Learning. IEEE Trans. Autom. Sci. Eng..

[10] Xiong X., Zheng K., Lei L., Hou L. (2020). Resource Allocation Based on Deep Reinforcement Learning in IoT Edge Computing. IEEE J. Sel. Areas Commun..

[11] Wang J., Zhao L., Liu J., Kato N. (2021). Smart Resource Allocation for Mobile Edge Computing: A Deep Reinforcement Learning Approach. IEEE Trans. Emerg. Top. Comput..

[12] Liu X., Yu J., Feng Z., Gao Y. (2020). Multi-agent reinforcement learning for resource allocation in IoT networks with edge computing. China Commun..

[13] Ren Y., Sun Y., Peng M. (2021). Deep Reinforcement Learning Based Computation Offloading in Fog Enabled Industrial Internet of Things. IEEE Trans. Ind. Inform..

[14] Cao Z., Zhou P., Li R., Huang S., Wu D.O. (2020). Multiagent Deep Reinforcement Learning for Joint Multichannel Access and Task Offloading of Mobile-Edge Computing in Industry 4.0. IEEE Internet Things J..

[15] Li T., Xu Z., Tang J., Wang Y. (2018). Model-Free Control for Distributed Stream Data Processing Using Deep Reinforcement Learning. Proc. VLDB Endow..

[16] Russo G.R., Nardelli M., Cardellini V., Presti F.L. (2018). Multi-Level Elasticity for Wide-Area Data Streaming Systems: A Reinforcement Learning Approach. Algorithms.

[17] Ye H., Li G.Y., Juang B.H.F. (2019). Deep Reinforcement Learning Based Resource Allocation for V2V Communications. IEEE Trans. Veh. Technol..

[18] Li Z., Guo C. (2020). Multi-Agent Deep Reinforcement Learning Based Spectrum Allocation for D2D Underlay Communications. IEEE Trans. Veh. Technol..

[19] Gong Y., Yao H., Wang J., Jiang L., Yu F.R. (2021). Multi-Agent Driven Resource Allocation and Interference Management for Deep Edge Networks. IEEE Trans. Veh. Technol..

[20] Murudkar C.V., Gitlin R.D. Optimal-Capacity, Shortest Path Routing in Self-Organizing 5G Networks using Machine Learning. Proceedings of the 2019 IEEE 20th Wireless and Microwave Technology Conference (WAMICON).

[21] Liu W., Cai J., Chen Q.C., Wang Y. (2021). DRL-R: Deep reinforcement learning approach for intelligent routing in software-defined data-center networks. J. Netw. Comput. Appl..

[22] Zhang W., Liu T., Xie M., Zhang J., Pan C. SAC: A Novel Multi-hop Routing Policy in Hybrid Distributed IoT System based on Multi-agent Reinforcement Learning. Proceedings of the 2021 22nd International Symposium on Quality Electronic Design (ISQED).

[23] You X., Li X., Xu Y., Feng H., Zhao J. Toward Packet Routing with Fully-distributed Multi-agent Deep Reinforcement Learning. Proceedings of the 2019 International Symposium on Modeling and Optimization in Mobile, Ad Hoc, and Wireless Networks (WiOPT).

[24] Ding R., Yang Y., Liu J., Li H., Gao F. Packet Routing Against Network Congestion: A Deep Multi-agent Reinforcement Learning Approach. Proceedings of the 2020 International Conference on Computing, Networking and Communications (ICNC).

[25] Mao Y., Zhang J., Letaief K.B. (2016). Dynamic Computation Offloading for Mobile-Edge Computing With Energy Harvesting Devices. IEEE J. Sel. Areas Commun..

[26] Jin T., Ji Z., Zhu S., Chen C. Learning-based Co-Design of Distributed Edge Sensing and Transmission for Industrial Cyber-Physical Systems. Proceedings of the 2021 IEEE 19th International Conference on Industrial Informatics (INDIN).

[27] Yang H., Alphones A., Zhong W.D., Chen C., Xie X. (2020). Learning-Based Energy-Efficient Resource Management by Heterogeneous RF/VLC for Ultra-Reliable Low-Latency Industrial IoT Networks. IEEE Trans. Ind. Inform..

[28] Wang X., Hu J., Lin H., Garg S., Kaddoum G., Jalilpiran M., Hossain M. (2021). QoS and Privacy-Aware Routing for 5G enabled Industrial Internet of Things: A Federated Reinforcement Learning Approach. IEEE Trans. Ind. Inform..

[29] Chen X., Hu J., Chen Z., Lin B., Xiong N., Min G. (2022). A Reinforcement Learning-Empowered Feedback Control System for Industrial Internet of Things. IEEE Trans. Ind. Inform..

[30] Sun P., Guo Z., Wang G., Lan J., Hu Y. (2020). MARVEL: Enabling controller load balancing in software-defined networks with multi-agent reinforcement learning. Comput. Netw..

[31] Li D., Tang H., Wang S., Liu C. (2017). A big data enabled load-balancing control for smart manufacturing of Industry 4.0. Cluster Comput..

[32] Zhang K., Yang Z., Basar T. (2019). Multi-Agent Reinforcement Learning: A Selective Overview of Theories and Algorithms. arXiv.

[33] Wiering M., van Otterlo M. (2012). Reinforcement Learning.

[34] Frochte J. (2018). Maschinelles Lernen.

[35] Bertsekas D.P. (2005). Dynamic Programming and Optimal Control.

[36] Shapley L.S. (1953). Stochastic Games. Proc. Natl. Acad. Sci. USA.

[37] Terry J.K., Black B., Hari A., Santos L., Dieffendahl C., Williams N.L., Lokesh Y., Horsch C., Ravi P. (2020). PettingZoo: Gym for Multi-Agent Reinforcement Learning. arXiv.

[38] Yang Y., Wang J. (2020). An Overview of Multi-Agent Reinforcement Learning from Game Theoretical Perspective. arXiv.

[39] Terry J.K., Grammel N., Black B., Hari A., Horsch C., Santos L. (2020). Agent Environment Cycle Games. arXiv.

[40] Wang H.N., Liu N., Zhang Y.Y., Feng D.W., Huang F., Li D.S., Zhang Y.M. (2020). Deep reinforcement learning: A survey. Front. Inf. Technol. Electron. Eng..

[41] Rosenberger J., Müller K., Selig A., Bühren M., Schramm D. Extended kernel density estimation for anomaly detection in streaming data. Proceedings of the 2021 15th CIRP Conference on Intelligent Computation in Manufacturing Engineering.

[42] Rauterberg F. (2022). Performance Vergleich von Datenkompressions Algorithmen Auf Industriellen Edge-Devices.

[43] Rosenberger J., Rauterberg F., Selig A., Bühren M., Schramm D. Perspective on Efficiency Enhancements in Processing Streaming Data in Industrial IoT Networks. Proceedings of the 2021 IEEE Global Conference on Artificial Intelligence and Internet of Things (GCAIoT) (2021 IEEE GCAIoT).

[44] Zaheer M., Kottur S., Ravanbakhsh S., Poczos B., Salakhutdinov R.R., Smola A.J., Guyon I., Luxburg U.V., Bengio S., Wallach H., Fergus R., Vishwanathan S., Garnett R. (2017). Deep Sets. Advances in Neural Information Processing Systems.

[45] Vinyals O., Fortunato M., Jaitly N., Cortes C., Lawrence N., Lee D., Sugiyama M., Garnett R. (2015). Pointer Networks. Advances in Neural Information Processing Systems.

[46] Vinyals O., Bengio S., Kudlur M. (2016). Order Matters: Sequence to sequence for sets. arXiv.

[47] Mao H., Gong Z., Xiao Z. (2020). Reward Design in Cooperative Multi-agent Reinforcement Learning for Packet Routing. arXiv.

[48] (2021). IEC 63278-1 ED1—Asset Administration Shell (AAS) for Industrial Applications—Part 1: Asset Administration Shell Structure.

[49] Hoffmeister M., Boss B., Orzelski A., Wagner J. (2021). Die Verwaltungsschale: Zentrum der digitalen Vernetzung in Fabriken (Teil 1). Atp Magazin.

[50] Alagha H.E. (2019). Communicating Intention in Decentralized Multi-Agent Multi-Objective Reinforcement Learning Systems. Master’s Thesis.

[51] https://www.gymlibrary.ml/.

[52] https://openai.com/.

[53] https://www.pettingzoo.ml/.

[54] https://stable-baselines3.readthedocs.io/en/master/.

